# Folic acid restricts SARS-CoV-2 invasion by methylating ACE2

**DOI:** 10.3389/fmicb.2022.980903

**Published:** 2022-08-17

**Authors:** Yuanzhou Zhang, Yechun Pang, Baiyin Xu, Xingshi Chen, Shunshun Liang, Jingying Hu, Xiaoying Luo

**Affiliations:** ^1^State Key Laboratory of Oncogenes and Related Genes, Shanghai Cancer Institute, Renji Hospital, Shanghai Jiao Tong University School of Medicine, Shanghai, China; ^2^Shanghai Pudong New Area People’s Hosptial, Shanghai, China

**Keywords:** SARS-CoV-2, methylation, ACE2, MTHFR, folic acid

## Abstract

The current COVID-19 pandemic is motivating us to elucidate the molecular mechanism of SARS-CoV-2 invasion and find methods for decreasing its transmissibility. We found that SARS-CoV-2 could increase the protein level of ACE2 in mice. Folic acid and 5-10-methylenetetrahydrofolate reductase (MTHFR) could promote the methylation of the ACE2 promoter and inhibit ACE2 expression. Folic acid treatment decreased the binding ability of Spike protein, pseudovirus and inactivated authentic SARS-CoV-2 to host cells. Thus, folic acid treatment could decrease SARS-CoV-2 invasion and SARS-CoV-2-neutralizing antibody production in mice. These data suggest that increased intake of folic acid may inhibit ACE2 expression and reduce the transmissibility of SARS-CoV-2. Folic acid could play an important role in SARS-CoV-2 infection prevention and control.

## Introduction

The coronavirus disease 2019 (COVID-19) pandemic caused by severe acute respiratory syndrome coronavirus 2 (SARS-CoV-2) has had disastrous consequences for public health, education, and the economy around the world (Gao et al., 2020; Chakraborty et al., 2021; Sun et al., 2021b). Coronaviruses mutate rapidly (Artese et al., 2020; Kadam et al., 2021), and the new mutations cause immune escape to a certain extent, which presents a challenge for the prevention and control of coronaviruses (Altmann et al., 2021; Chmielewska et al., 2021; Callaway, 2021b; Wang et al., 2021b; The Lancet Infectious Diseases, 2022). According to a mathematical model (Bushman et al., 2021), SARS-CoV-2 variants will continue spreading with enhanced transmissibility and partial immune escape, even as immunity accumulates in the population, which will limit the impact of vaccination and exacerbate the pandemic. Therefore, the key issue is to decrease transmissibility and immune escape.

Vaccination is an effective method to prevent the immune escape of viruses (Weisblum et al., 2020; Zhang et al., 2021). Neutralizing antibody levels are highly predictive of immune protection from symptomatic SARS-CoV-2 infection (Bushman et al., 2021). The release of COVID-19 vaccines, such as mRNA vaccines (Pfizer and Moderna), inactivated virus vaccines (Sinovac, Sinopharm), and adenovirus vector vaccines (AstraZeneca, CanSino Biologics, Gamaleya Research Institute and J&J), has helped decrease the immune escape of the virus (Chung et al., 2021; Soiza et al., 2021). However, there is growing evidence that vaccines may be less protective against new variants, and the possibility of immune escape is increasing (Forni and Mantovani, 2021; Callaway, 2021a).

As an RNA virus, SARS-CoV-2 is more prone to mutation than DNA viruses such as hepatitis B virus (Covid C. D. C. and R Team, 2021; Grint et al., 2021; Levine-Tiefenbrun et al., 2021; Rubin et al., 2021; Zhang et al., 2021). The more worrisome fact is that the COVID-19 pandemic is providing the virus with a ‘breeding ground’ for mutations. Development of vaccine for the new variant of a particular need time and money, and the mutation of high frequency for vaccine development challenges in a timely manner (Zhou and Wang, 2021). Therefore, a convenient method may be needed to safely and effectively increase neutralizing antibody production for existing vaccines.

With the revelation that ACE2 is the target of SARS-CoV-2 invading the human body, blocking the binding of SARS-CoV-2 and ACE2 has also become a therapeutic direction (Delorme-Axford and Klionsky, 2020; Tada et al., 2020; Vandelli et al., 2020; Oz et al., 2021; Wang et al., 2021a). SARS-CoV-2 transmissibility is mainly based on the strength of the binding between SARS-CoV-2 and the ACE2 protein (Huang et al., 2021; Oz et al., 2021). Scientists have made many efforts to reduce the binding between the ACE2 protein and SARS-CoV-2: polysulfates have been used to block the electrostatic interaction near the ACE2 binding site of SARS-CoV-2 (Nie et al., 2021), aptamers have been designed to block the interaction between the receptor-binding domain (RBD) of SARS-CoV-2 and host ACE2 (Sun et al., 2021a), and ACE2-modified extracellular vesicles have been designed to compete with host cell ACE2 for binding to SARS-CoV-2 (Kim et al., 2022). However, there is much more work to be done prior to the clinical application of the above methods to reduce virus transmissibility. The ACE2 protein plays a vital role in cardiovascular diseases/nephropathy (Santos et al., 2018; Evans et al., 2020; Gheblawi et al., 2020; Zheng et al., 2020). Some ACE2 inhibitors are used clinically. However, these ACE2 inhibitors are not applicable in healthy people (Bavishi et al., 2020; Cao et al., 2020; Vaduganathan et al., 2020; South et al., 2020b). Complications such as hypertension are key factors in determining the prognosis of patients, as is the case for COVID-19 (Chinese case report: Among 1,099 patients during the COVID-19 pandemic, hypertension was the most common complication, with an estimated prevalence rate of 15%; Xiong et al., 2020). Decreasing the level of ACE2 will cause an imbalance between the ACE/AngII/AT1R and ACE2/Ang1-7/MasR axes, resulting in vasoconstriction and inflammatory effects, negatively affecting the progression of the disease (Hatmal et al., 2020; Oz et al., 2021; Gintoni et al., 2022). Therefore, it is important to find a method that is safe and effective for healthy people.

In this work, we found that MTHFR overexpression or folic acid treatment could decrease the ACE2 protein level by methylation of the ACE2 promoter. Folic acid reduced SARS-CoV-2 binding to cells, SARS-CoV-2 infection and neutralizing antibody production. Folic acid is a food additive (Bae et al., 2021; Roche et al., 2021; Shulpekova et al., 2021) that may be a convenient, safe, and effective way to reduce SARS-CoV-2 transmissibility; on the other hand, diminished folic acid levels in food may increase neutralizing antibody production. This strategy may provide a safe and autonomously regulated means of controlling virus transmissibility and immune escape.

## Materials and methods

### Inactivated virus preparation

All experiments with live SARS-CoV-2 were performed in enhanced biosafety level 3 (P3+) facilities. SARS-CoV-2 was provided by Sinovac Biotech (Beijing, China) and inactivated with β-propiolactone for 24 h.

### *In vivo* experiments

The 6-week BALB/c mice were assigned to 2 groups for tail vein injection: the inactivated SARS-CoV-2 group (3 μg/dose) and the saline control group. The injections were given three times per week for 3 weeks. Heart, liver, spleen, lung, kidney, brain tissue and plasma were collected 21 days after inoculation for haematological analysis and SARS-CoV-2-neutralizing antibody detection. Folic acid treatment experimental animals were assigned to two groups for intraperitoneal injection, the folic acid (50 mg/kg) and saline control group, which were injected twice per week for a total of 5 weeks. After 2 weeks of folic acid pretreatment, both groups were given the above tail vein injection to inactivate SARS-CoV-2. Heart, liver, spleen, lung, kidney, brain tissue and plasma were collected after 5 weeks for haematological analysis and SARS-CoV-2-neutralizing antibody detection.

### Cell culture and reagents

HEK293T, PANC02, HEPG2 and Huh7 human CRC cell lines were purchased from the American Type Culture Collection (ATCC, Rockville, MD, United States). HEK293T, PANC02, HEPG2 and Huh7 cells were maintained in DMEM (Gibco, Grand Island, NY, United States). The medium was supplemented with 10% foetal bovine serum, and the cells were incubated in a humidified atmosphere of 95% air and 5% CO_2_ at 37°C.

### Transfection

Lentiviruses containing MTHFR or a control were purchased from GeneChem (Shanghai, China), and the transfection of lentivirus into CRC cells was performed according to the manufacturer’s protocol. After lentivirus infection, monoclonal cells were selected and cultured to check for the expression of MTHFR by qRT–PCR. MTHFR siRNA was synthesized by GenePharma (Shanghai, China) and transfected into cells using Lipofectamine™ 2000 (Invitrogen, United States) according to the manufacturer’s protocol.

### RT–PCR (Reverse Transcription-Polymerase Chain Reaction)

Total RNA was extracted from organs with an RNeasy Mini Kit (Qiagen) and a PrimerScript RT Reagent Kit (TaKaRa). The forward and reverse primers targeting the envelope (E) gene of SARS-CoV-2 used for RT–PCR were 5′- TCGTTTCGGAAGAGACAGGT-3′ and 5′- GCGCAGTAAGGATGGCTAGT-3′, respectively. RT–PCR was performed under the reaction conditions of 50°C for 30 min, followed by 40 cycles of 95°C for 15 s, 94°C for 15 s, and 60°C for 45 s.

### DNA extraction and methylation analysis

Genomic DNA was isolated from Huh-7 cells using a DNA isolation kit (Tiangen, Beijing, China) following the manufacturer’s protocol. Bisulfite conversion was measured using the EpiTect Bisulfite Kit (Qiagen). Methylation levels of ACE2 were analysed by methylation-specific PCR. The ACE2 repeat sequence primer sequences were as follows: forward: 5′-TTAGTGAATTTTTAATGGATTAGAGATATA-3′; reverse: 5′-TTTATCACCCAAACTAAAATACAAC-3′. The bisulfite sequencing PCR system consisted of 10 × PCR buffer and 5.25 μm dNTP Mix, 0.5 μm each primer, 0.75 U of hot-start DNA polymerase, and 20 ng of bisulfite-modified DNA. The bisulfite PCR products were analysed on a 2% agarose gel and purified using a QIAquick gel extraction kit (Qiagen). Each purified product was cloned into a pMD 19-T vector (Takara, Tokyo, Japan) and then transfected into *E. coli* DH5α competent cells (Vazyme Biotech Co., Piscataway, NJ, United States). For each product, five clones were sequenced (Shanghai Xinren Molecular Biotechnology Co., Ltd., China).

### Antibodies and western blotting

Protein aliquots of 25 μg each were resolved by SDS polyacrylamide gel electrophoresis (Bio-Rad, Hercules, CA) and detected with the appropriate antibodies. Electrophoresed protein samples were transferred to polyvinylidene difluoride membranes (Bio-Rad). After being washed three times, the membranes were incubated in blotting-grade blocker (Bio-Rad) for 1 h at room temperature and then overnight at 4°C with primary antibodies specific to MTHFR (Cell Signaling Technology, Danvers, MA, United States), ACE2 (Abcam, Cambridge, MA, United States) and β-actin (CST). After being washed three times, the membranes were incubated for 1 h at room temperature with an HRP-conjugated species-specific secondary antibody. Immunoreactive bands were visualized using SuperSignal West Dura Extended Duration Substrate Enhanced Chemiluminescent Substrate (Pierce Biotechnology, Rockford, IL, United States). Each experiment was independently performed at least three times.

### Analysis of the SARS-CoV-2 spike protein, pseudovirus and inactivated virus combination using confocal microscopy

HEK293T, PANC02, and Huh7 cells (5 × 10^4^) were seeded onto cover glass in 6-well plates. After 24 h, the cell cultures were replenished with DMEM and incubated with Spike protein (Sinobiological, Beijing, China), pseudovirus (Sinobiological, Beijing, China) or inactivated virus in a 5% CO2 incubator for 30 min at 37°C. The cells were fixed with 4% paraformaldehyde and subsequently blocked and permeabilized with phosphate-buffered saline with Tween-20 (PBST) containing 1% bovine serum albumin (BSA). Since the Spike protein and pseudovirus carried a His-tag, we incubated the cultures with an anti-His primary antibody overnight. The inactivated virus was incubated overnight with a Spike-neutralizing antibody. Alexa Fluor-488-conjugated streptavidin (Boster, Beijing, China) was added, and the cultures were incubated for 1 h. Then, the samples were washed with PBST, and Hoechst 33258 (Thermo Fisher Scientific, Waltham, MA, United States) was added to stain the nuclei. The slides were analysed by confocal microscopy using a TCS SP8 microscope (Leica, Germany).

### 50% effective concentration analysis

HEK293T, PANC02, and Huh7 cells (4 × 10^3^) were seeded in 96-well plates. The immunofluorescence staining procedure was the same as that for the laser confocal experiment. Celigo image cytometry (Nexcelom Bioscience, United States) was used to complete quantitative single-cell fluorescence detection. According to the fluorescence signal, the inhibition rate of the host cells at the concentration of Spike protein, pseudovirus or inactivated virus was calculated. Taking the concentration of Spike protein, pseudovirus or inactivated virus as the abscissa, and the inhibition rate of cells as the ordinate, the dose-dependent curve of the inhibition rate of cell binding was drawn by Graphpad 8.0, and the regression was performed to calculate the 50% effective concentration.

### Fluorescence quantitative analysis

The immunofluorescence staining procedure was the same as that for the laser confocal experiment. The cells were seeded into a 96-well plate, and Celigo image cytometry (Nexcelom Bioscience, United States) was used to complete quantitative single-cell fluorescence detection.

### Enzyme-linked immunosorbent assay

The SARS-CoV-2 antibody titre of serum samples collected from immunized BALB/c mice was determined by indirect ELISA (Sinobiological, Beijing, China). Diluted sera (1,100) were applied to each well for 2 h at 37°C according to the instructions. Finally, a microplate reader was used to read the absorbance value at 450 nm. An ACE2 mouse ELISA kit was purchased from Sangon Biotech (Shanghai, China), and the plasma samples were added according to the instructions. The absorbance (OD) value of the reaction well samples was measured at 450 nm.

## Results

### SARS-CoV-2 exposure resulted in elevated ACE2

**Figure 1:**
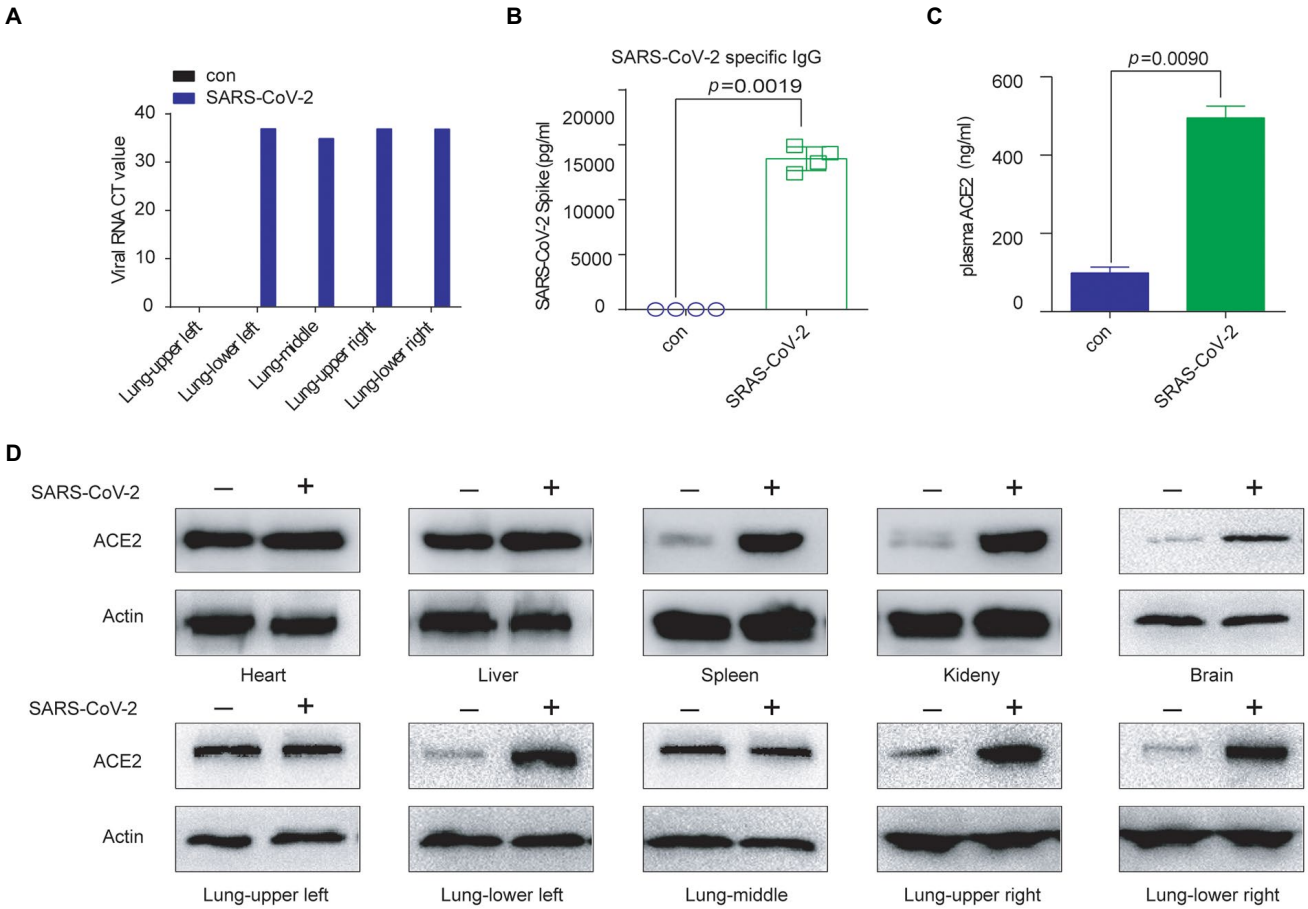
SARS-CoV-2 exposure resulted in elevated ACE2. **(A)** The SARS-CoV-2 RNA level was increased in the mouse lung (except the upper-left part) by inactivated virus treatment. **(B)** SARS-CoV-2–specific neutralizing antibody levels were increased in BALB/c mice treated with inactivated virus, as detected by ELISA. **(C)** The ACE2 protein level was increased in BALB/c plasma by inactivated virus treatment, as detected by ELISA. **(D)** The expression of ACE2 protein was significantly upregulated in the main organs by inactivated SARS-CoV-2 treatment, except in the middle and upper-left lung.

First, we validated the invasion efficiency of SARS-CoV-2 in the BALB/c mice. After inactivated SARS-CoV-2 treatment of BALB/c mice *via* the tail vein, we found that the SARS-CoV-2 RNA level was increased in all lung regions except the upper left lobe (Figure 1A), and SARS-CoV-2–specific neutralizing antibody levels were also increased in mouse plasma (Figure 1B).

At the same time, we detected elevated levels of ACE2 in plasma (Figure 1C). Then, we checked ACE2 expression in the main organs of the mice. The change in ACE2 mRNA levels was not consistent. The ACE2 mRNA level increased significantly in the spleen, left lower lung, right upper lung and right upper lung (Supplementary Figure 1A). However, the ACE2 protein level increased consistently in the main organs of inactivated SARS-CoV-2-treated mice (did not change in the upper-left and middle lungs), including in the spleen, where neutralizing antibodies are produced (Figure 1D).

### The folic acid system affects the expression of ACE2 by regulating methylation in the promoter region of ACE2

**Figure 2:**
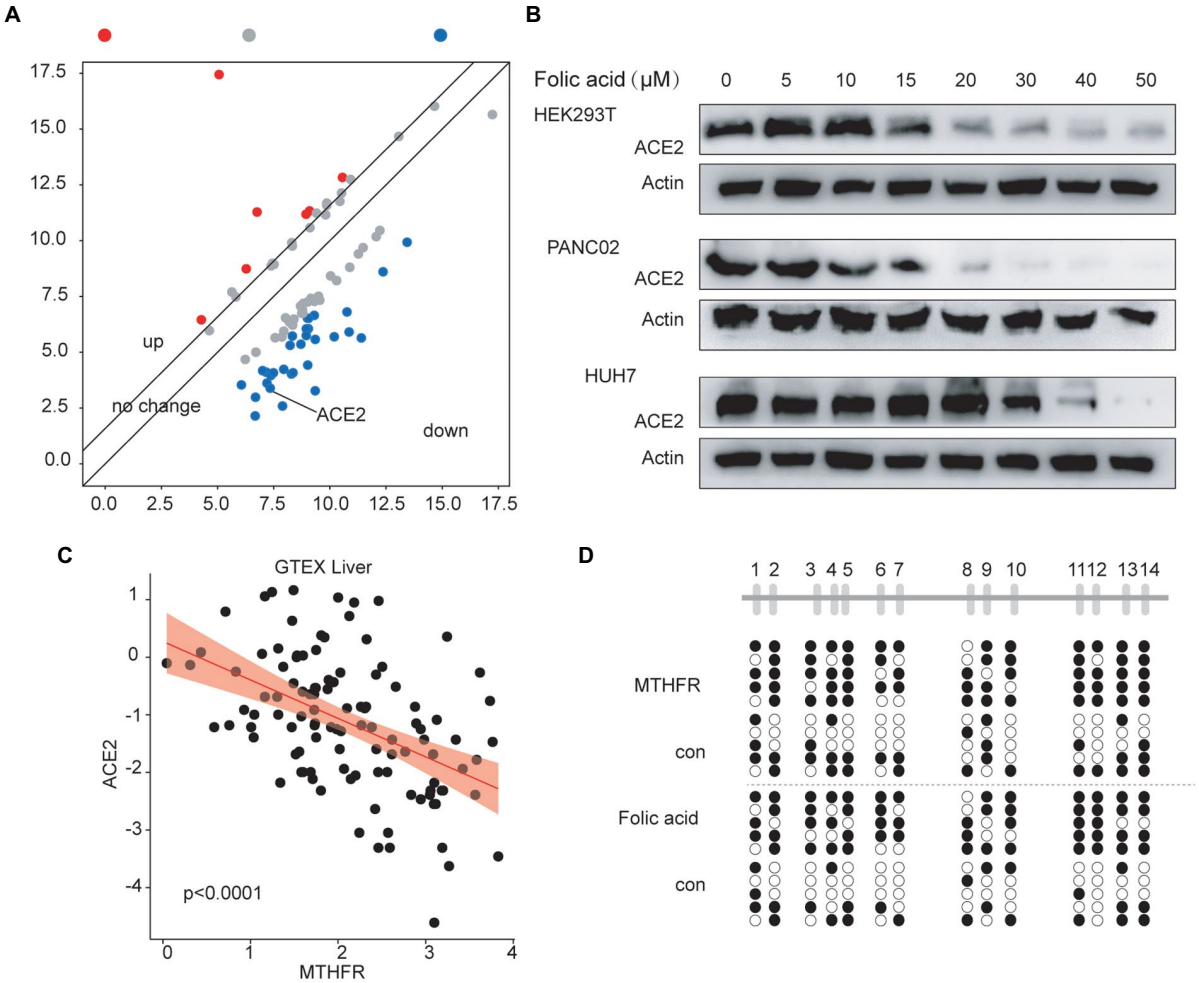
The Folic acid system affects the expression of ACE2 by regulating methylation in the promoter region of ACE2. **(A)** The ACE2 protein level was decreased in MTHFR-overexpressing Huh7 cells, as detected by an antibody microarray assay. **(B)** Folic acid inhibited ACE2 protein expression in a dose-dependent manner in HEK293T, PANC02, and Huh7 cells. **(C)** MTHFR expression was negatively correlated with ACE2 expression (liver in the GTEX database, liver cancer in the TCGA database). **(D)** MTHFR overexpression promoted ACE2 promoter DNA methylation in Huh7 cells. Folic acid promoted ACE2 promoter DNA methylation in Huh7 cells.

We found that the ACE2 protein level was decreased in Huh7 cells stably overexpressing MTHFR (Figure 2A), as detected by a Human Cytokine Array Q440. We further analysed the correlation between ACE2 and MTHFR in normal liver tissues in the GTEX database and liver cancer tissues in the TCGA database, and the results showed that ACE2 and MTHFR were negatively correlated (Figure 2C; Supplementary Figure 1B). The ACE2 protein level was significantly reduced in MTHFR-overexpressing Huh7 and HepG2 cells (Supplementary Figures 1C–F).

Then, we detected changes in ACE2 protein levels after treatment with a concentration gradient of folic acid in the HEK293T, PANC02 and Huh7 cell lines. These results showed that folic acid decreased the expression of ACE2 in a dose-dependent manner (Figure 2B). We also found that MTHFR protein levels continued to decrease in the major organs of inactivated SARS-CoV-2-treated mice (Supplementary Figure 1G).

We further speculate that folic acid decrease protein level of ACE2 through the MTHFR-DNA methylation regulatory mechanism. Therefore, we detected the DNA methylation level of the ACE2 promoter in MTHFR-overexpressing cells or folic acid-treated cells. The data showed that MTHFR overexpression and folic acid treatment induced a increase in ACE2 promoter DNA methylation levels (Figure 2D).

### Folic acid treatment reduces the spike protein binding ability

**Figure 3:**
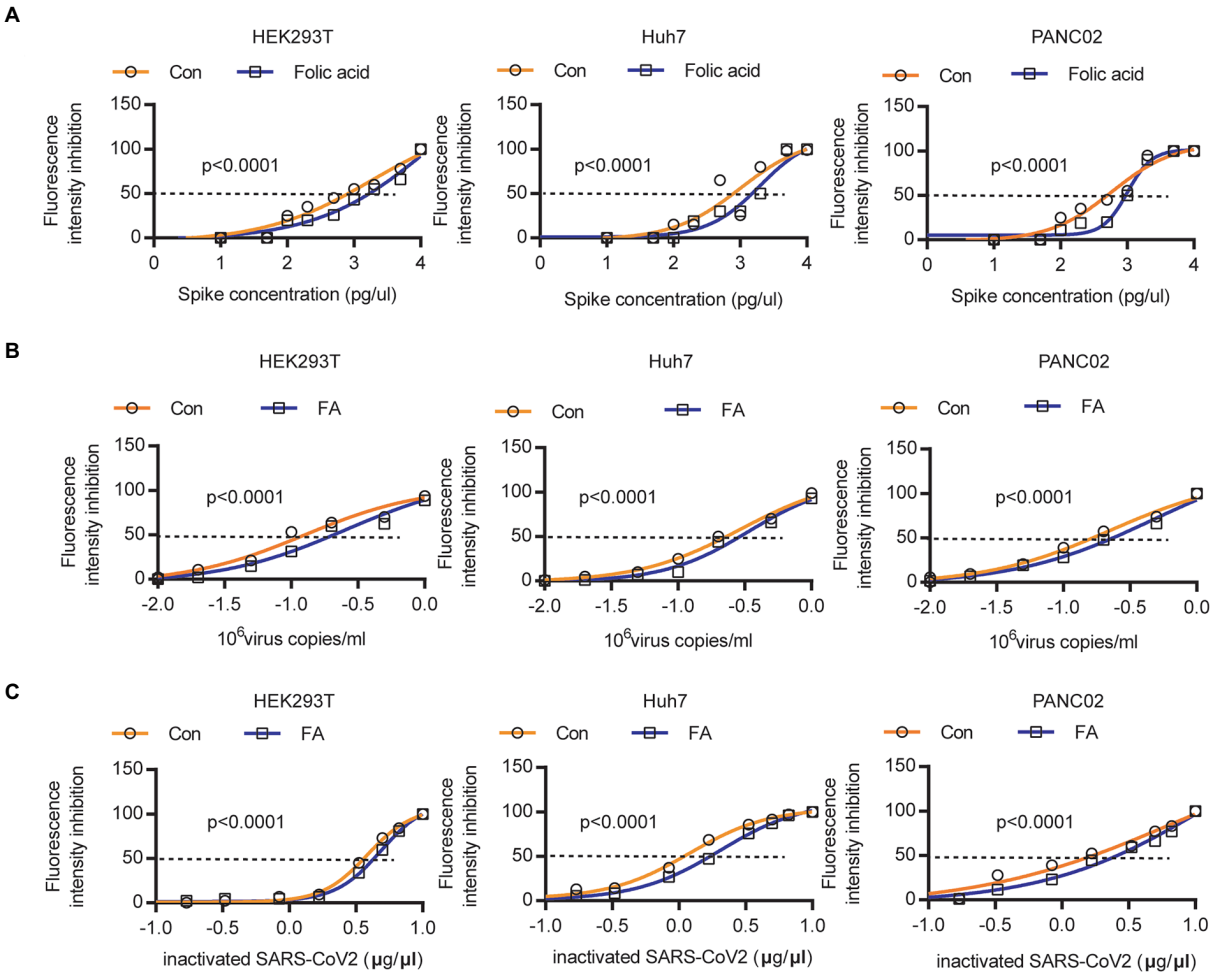
Folic acid reduced the 50% Spike protein or SARS-CoV-2 binding to host cells. **(A)** The 50% effective concentration of Spike protein was increased by 61.6, 98.2 and 72.1% in HEK293T/PANC02/Huh7 cells. **(B)** The 50% effective concentration of pseudoviruses was increased by 93.2, 15.3 and 93.1% in HEK293T/PANC02/Huh7 cells. **(C)** The 50% effective concentration of inactivated authentic SARS-CoV-2 was increased by 17.6, 65.3 and 70.8% in HEK293T/PANC02/Huh7 cells.

We validated that folic acid or MTHFR could inhibit ACE2 protein expression by methylating the ACE2 promoter. Then, we detected the influence of folic acid treatment on the 50% effective concentration of Spike protein. After folic acid treatment, the 50% effective concentration of SPIKE protein in HEK293T/PANC02/Huh7 cells increased by 61.6, 98.2 and 72.1%, respectively (Figure 3A). Correspondingly, the 50% effective concentrations of pseudoviruses increased by 93.2, 15.3 and 93.1% (Figure 3B), while the 50% effective concentrations of inactivated authentic SARS-CoV-2 increased by 17.6, 65.3 and 70.8% (Figure 3C).

**Figure 4:**
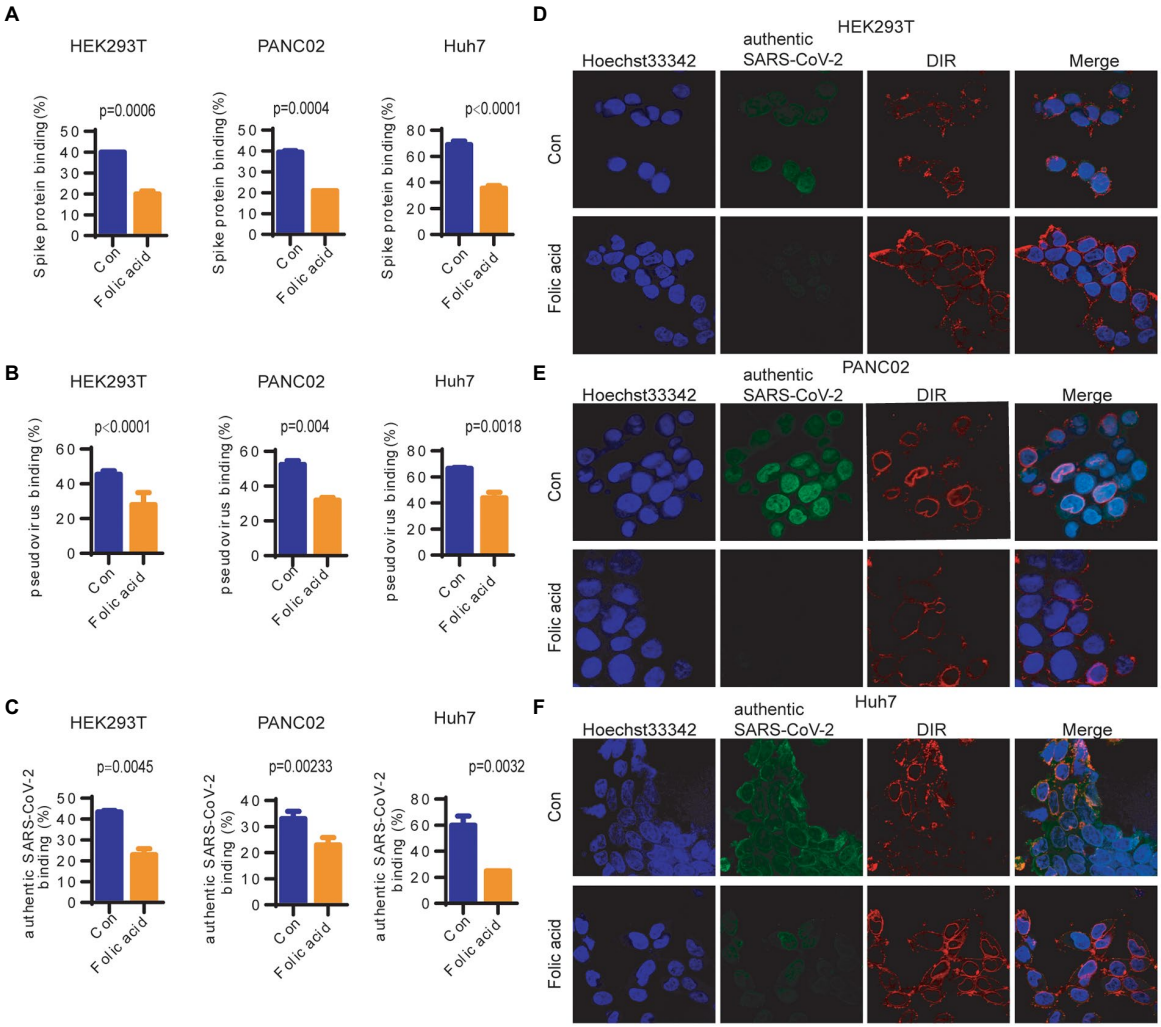
The binding ability of SARS-CoV-2 to cells was reduced by folic acid treatment. **(A)** The binding ability of the Spike protein to cells was reduced to 47.5%/53.8%/50.7% in HEK293T/PANC02/Huh7 cells at a 15,000 pg./μL Spike protein concentration. **(B)** The binding ability of SARS-CoV-2 pseudovirus to cells was reduced to 52.2%/39.2%/29.8% in HEK293T/PANC02/Huh7 cells at 1.5 × 10^6^ pseudovirus copies/ml. **(C)** The binding ability of inactivated authentic SARS-CoV-2 to cells was reduced to 52.2%/29.3%/54.5% in HEK293T/PANC02/Huh7 cells at 10 μg/μl inactivated SARS-CoV-2. The representative image shows that the binding ability of inactivated authentic SARS-CoV-2 to cells was reduced by folic acid treatment, as detected by confocal microscopy in HEK293T **(D)** PANC02 **(E)** and Huh7 **(F)** cells. Cells were treated with 20 μm folic acid for 48 h, and different concentrations of authentic SARS-CoV-2 were added to the medium and allowed to bind for 1 h. The quantification of binding ability was detected by Celigo assay. The cell membrane was labelled with DiR (red), the spike protein and pseudovirus/authentic SARS-CoV-2 were labelled with an anti-spike antibody (green), and the cell nucleus was labelled with Hoechst (blue). The statistical analysis of virus binding (the fluorescence intensity of Spike protein on cells, Hoechst used as internal control) differences caused by folic acid treatment was performed by two-way ANOVA.

After treatment with 20 μm folic acid for 48 h, different concentrations of the Spike protein were added to the medium. At a Spike protein concentration of 15,000 pg./μL, the Spike protein binding ability was reduced to 47.5%/53.8%/50.7% in HEK293T/PANC02/Huh7 cells (Figure 4A; Supplementary Figure 2). At a concentration of 1.5 × 10^6^ pseudovirus copies/ml, the pseudovirus binding ability was reduced to 52.2%/39.2%/29.8% in HEK293T/PANC02/Huh7 cells (Figure 4B; Supplementary Figure 3). At a 10 μg/μl inactivated SARS-CoV-2 concentration, the inactivated authentic SARS-CoV-2 binding ability was reduced to 52.2%/29.3%/54.5% in HEK293T/PANC02/Huh7 cells (Figure 4C). A representative image showed that the binding ability of inactivated authentic SARS-CoV-2 to HEK293T (Figure 4D)/PANC02 (Figure 4E)/Huh7 (Figure 4F) cells was reduced by folic acid treatment. Therefore, SARS-CoV-2 binding to cells was decreased significantly by folic acid treatment.

### Folic acid treatment inhibited SARS-CoV-2 invasion and neutralizing antibody production *in vivo*

**Figure 5:**
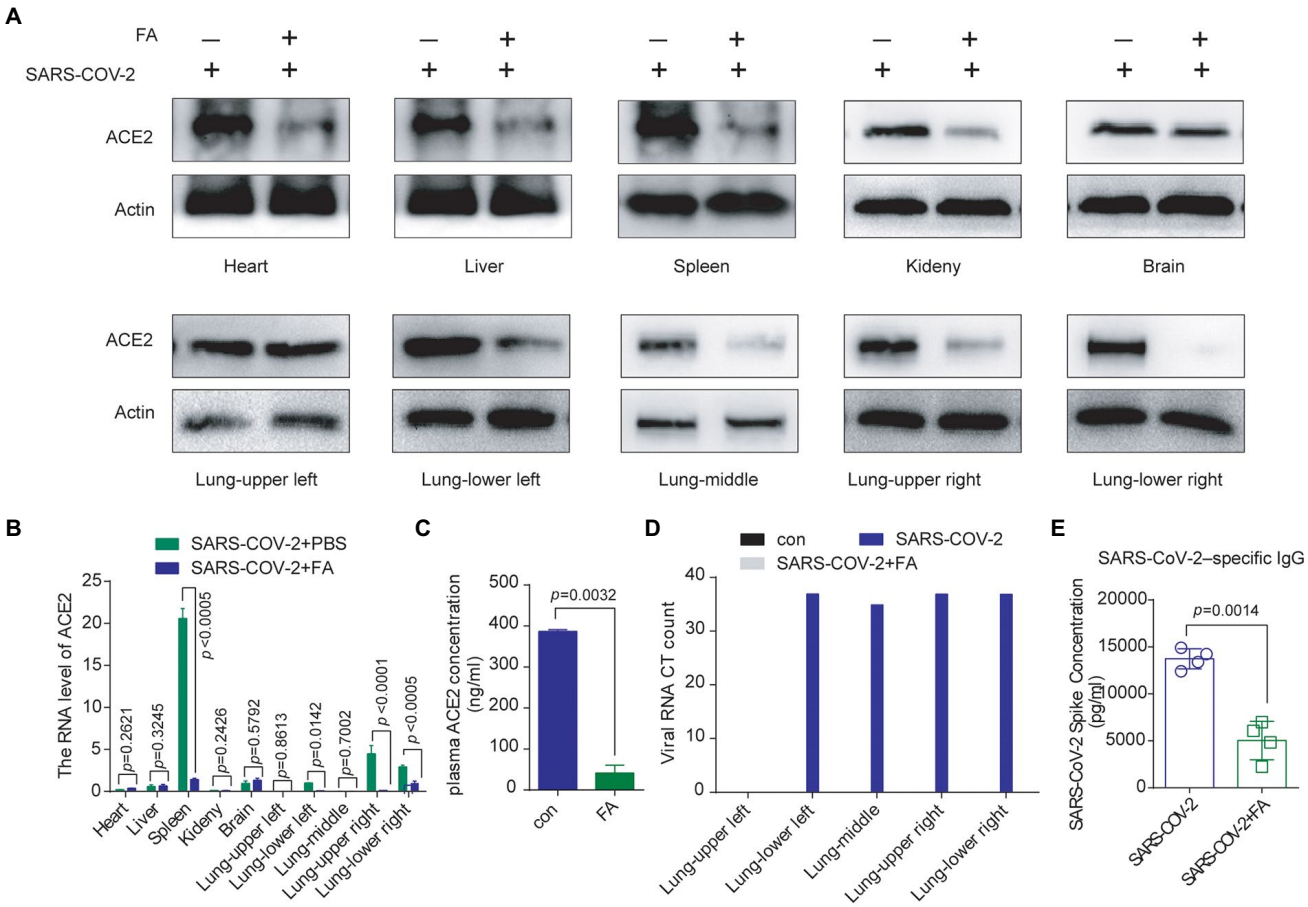
Folic acid treatment decreased ACE2 expression and inhibited SARS-CoV-2 invasion and SARS-CoV-2-neutralizing antibody production *in vivo*. **(A)** Folic acid treatment decreased ACE2 protein expression in the main organs of BALB/c mice. **(B)** The ACE2 mRNA level decreased in the main organs of folic acid-treated BALB/c mice, as examined by a qRT–PCR assay. **(C)** The plasma ACE2 protein concentration was decreased in folic acid-treated BALB/c mice, as detected by ELISA. **(D)** Folic acid treatment decreased SARS-CoV-2 invasion, and SARS-CoV-2 RNA levels were decreased in each part of the BALB/c mouse lung. **(E)** SARS-CoV-2–specific neutralizing antibody levels were decreased in folic acid-treated BALB/c mice, as detected by ELISA.

Then, we checked whether folic acid influences SARS-CoV-2 invasion. After pretreatment with folic acid for 2 weeks, treatment with inactivated authentic SARS-CoV-2, and treatment with folic acid for another 2 weeks, we checked SARS-CoV-2 invasion and neutralizing antibody production. We found that the ACE2 protein level decreased in the main organs, including in the spleen, where neutralizing antibodies are produced (Figure 5A, except for the upper left part of the lung), and in the plasma (Figure 5C) of folic acid-treated BALB/c mice. The ACE2 mRNA level decreased in the main organs (Figure 5B, except for the upper left and middle parts of the lung). Then, viral invasion in the lungs was almost cleared by folic acid treatment (Figure 5D), and neutralizing antibody production decreased to 36.7% (compared to that in the folic acid untreated control; Figure 5E).

## Discussion

In this study, we found that SARS-CoV-2 exposure resulted in elevated ACE2. After the administration of folic acid, the protein level of ACE2 in the tissue decreased, resulting in the removal of the authentic SARS-CoV2 in the lung tissue, concomitantly decreased levels of sars-cov-2 neutralizing antibodies.

ACE2 plays a balancing role in blood pressure regulation. Guillaume Paré’s team reported that an increased plasma ACE2 concentration was significantly associated with an increased risk of major cardiovascular events (Narula et al., 2020). An increased plasma ACE2 concentration is also significantly associated with an increased risk of heart failure, myocardial infarction, stroke, and diabetes (Aleksova et al., 2020; Jia et al., 2020; South et al., 2020a). Although our results showed different levels of ACE2 mRNA in various organs of the mice, the protein levels were significantly elevated, which may explain the high ACE2 expression associated with the high incidence of cardiovascular complications after SARS-CoV-2 stimulation.

MTHFR is a key enzyme in the folate circulation system (Christensen et al., 2015; Moll and Varga, 2015; Roman et al., 2019; Weile et al., 2021). Accelerating folate circulation can increase the activity of MTHFR or directly enrich folic acid, suggesting that folic acid supplementation may regulate ACE2 expression. Lack of MTHFR expression causes elevated homocysteine levels, which in turn leads to an increased risk of thrombosis and is associated with a higher risk of cardiovascular disease (Raghubeer and Matsha, 2021). This may explain why people with underlying cardiovascular disease tend to become sicker after contracting a respiratory virus. Early case reports of novel coronavirus pneumonia showed that up to 50% of patients had underlying diseases, and 40% of them had cardiovascular or cerebrovascular diseases (Xiong et al., 2020). There have been clinical studies reporting newly diagnosed cases of cerebral sinus vein thrombosis in patients with MTHFR mutations following injection of the vector-based ChAdOx1 COVID-19 vaccine (Fousse et al., 2022).

Folic acid can minimize plasma homocysteine concentrations, thereby reducing risk factors for vascular disease. In our work, folic acid can reduce virus neutralizing antibodies and to a certain extent reduce the probability of virus binding. Based on the effect of folic acid on the expression of ACE2, it may increase benign benefits for cardiovascular diseases. Therefore, in this study, the significance of folic acid is not only to reduce the expression of ACE2 to reduce the invasion of SARS-CoV-2 but also to have a beneficial impact on the complications of cardiovascular disease caused by SARS-CoV-2. In short, as a health food additive approved by the FDA, folic acid can reduce virus infection and the production of neutralizing antibodies to a certain extent, suggesting its important role in epidemic prevention.

## Conclusion

**Figure 6:**
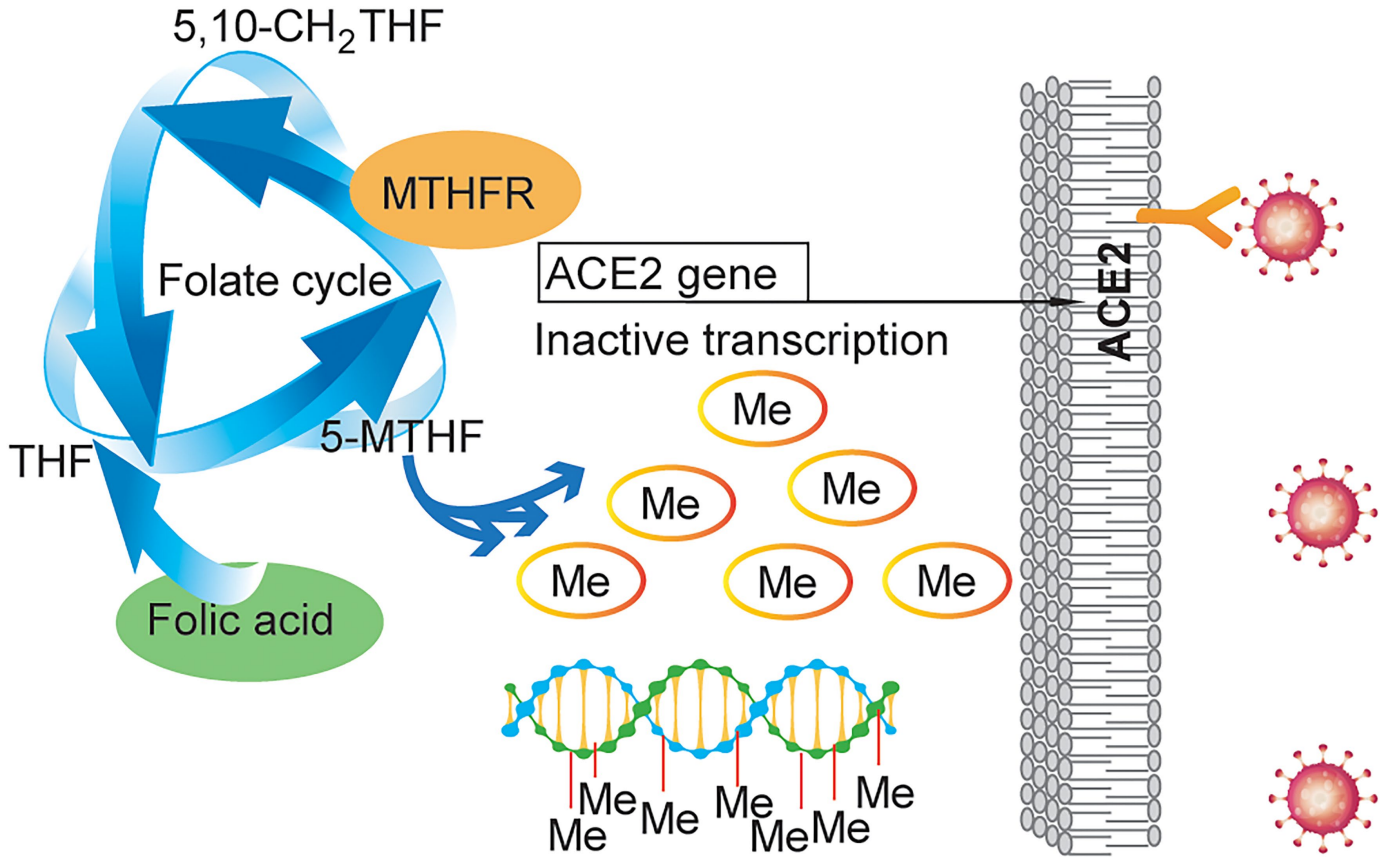
Schematic illustrating the effect of folic acid on SARS-CoV-2 invasion. MTHFR regulates ACE2 expression by changing its promoter methylation level, and folic acid can alter SARS-CoV-2 transmissibility and SARS-CoV-2-neutralizing antibody production.

In summary, we demonstrated that SARS-CoV-2 could reduce the expression of MTHFR and increase the expression of ACE2, that MTHFR could regulate ACE2 expression by changing its promoter methylation level, and that folic acid could alter SARS-CoV-2 transmissibility/neutralizing antibody production (Figure 6).

As an FDA-approved food additive (Field and Stover, 2018), increased intake of folic acid may inhibit ACE2 expression and reduce SARS-CoV-2 transmissibility, and limiting folic acid intake to increase ACE2 expression before vaccination may increase neutralizing antibody production. These data suggest that folic acid may play a role in SARS-CoV-2 infection prevention and control.

## Data availability statement

The original contributions presented in the study are included in the article/Supplementary material, further inquiries can be directed to the corresponding author.

## Ethics statement

The animal study was reviewed and approved by Institutional Animal Care and Use Committee of Model Animal Research Center of Shanghai Cancer Institute.

## Author contributions

XL: manuscript editing, review, and data analysis. YZ, YP, and BX: data collection and experiments. XC, SL, and JH: figures preparation. All authors contributed to the article and approved the submitted version.

## Funding

The study was supported by National key research and development program (2022YFE0110100) and the Natural Science Foundation of China (81572454).

## Conflict of interest

The authors declare that the research was conducted in the absence of any commercial or financial relationships that could be construed as a potential conflict of interest.

## Publisher’s note

All claims expressed in this article are solely those of the authors and do not necessarily represent those of their affiliated organizations, or those of the publisher, the editors and the reviewers. Any product that may be evaluated in this article, or claim that may be made by its manufacturer, is not guaranteed or endorsed by the publisher.
