# Supplementary material for: Folic acid restricts SARS-CoV-2 invasion by methylating ACE2
Source: Front Microbiol. 2022 Aug 17;13:980903. doi: 10.3389/fmicb.2022.980903 (PMC9432853; doi:10.3389/fmicb.2022.980903)
Supplement: Supplementary file 1 [file Data_Sheet_1.pdf]

Supplementary Materials

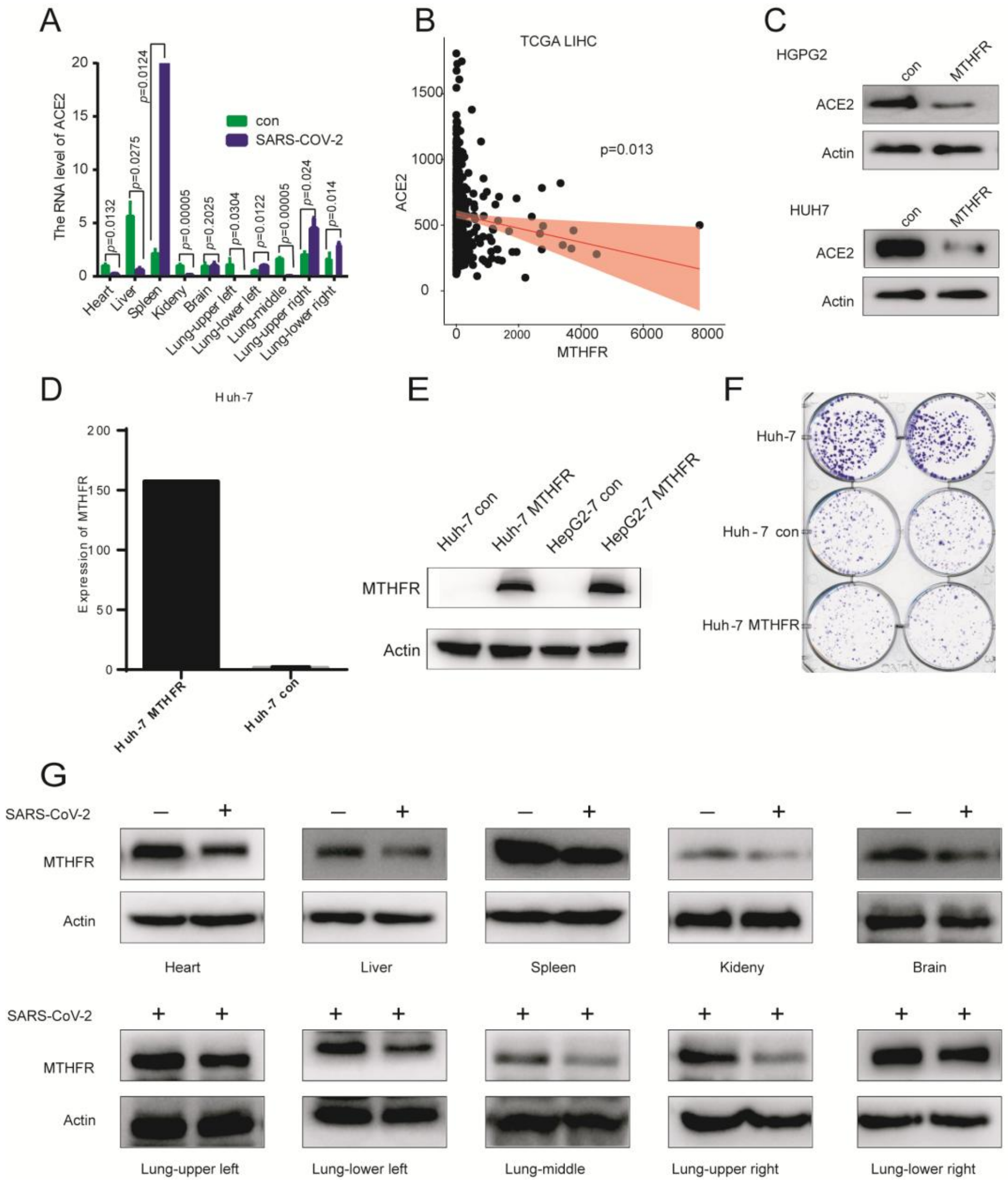

Supplementary Figure **1**. (A) ACE2 mRNA expression in different organs was examined by qRT–PCR. (B) MTHFR expression was negatively correlated with ACE2 expression (TCGA data). (C) ACE2 protein levels in MTHFR-overexpressing cells were detected by western blotting. (D) Verification of MTHFR mRNA levels by qRT–PCR after lentivirus transfection was to create a stable MTHFR-overexpressing cell line. (E) Verification of MTHFR protein expression by western blotting after lentivirus transfection was to create a stable MTHFR-overexpressing cell line. (F) Colony formation assay after lentivirus transfection was to create a stable MTHFR-overexpressing cell line. (G) The expression of MTHFR protein was significantly downregulated in the main organs by inactivated SARS-CoV-2 treatment.

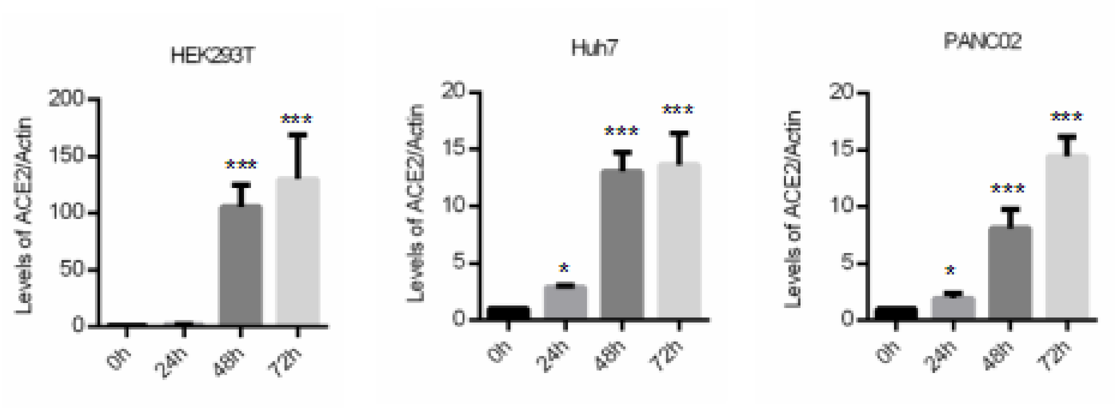

Supplementary Figure 2. The mRNA levels of ACE2 were detected by rt-qPCR after 5-aza-dC treatment. *$p < 0.05$, ***$p < 0.001$, compared to untreated samples.

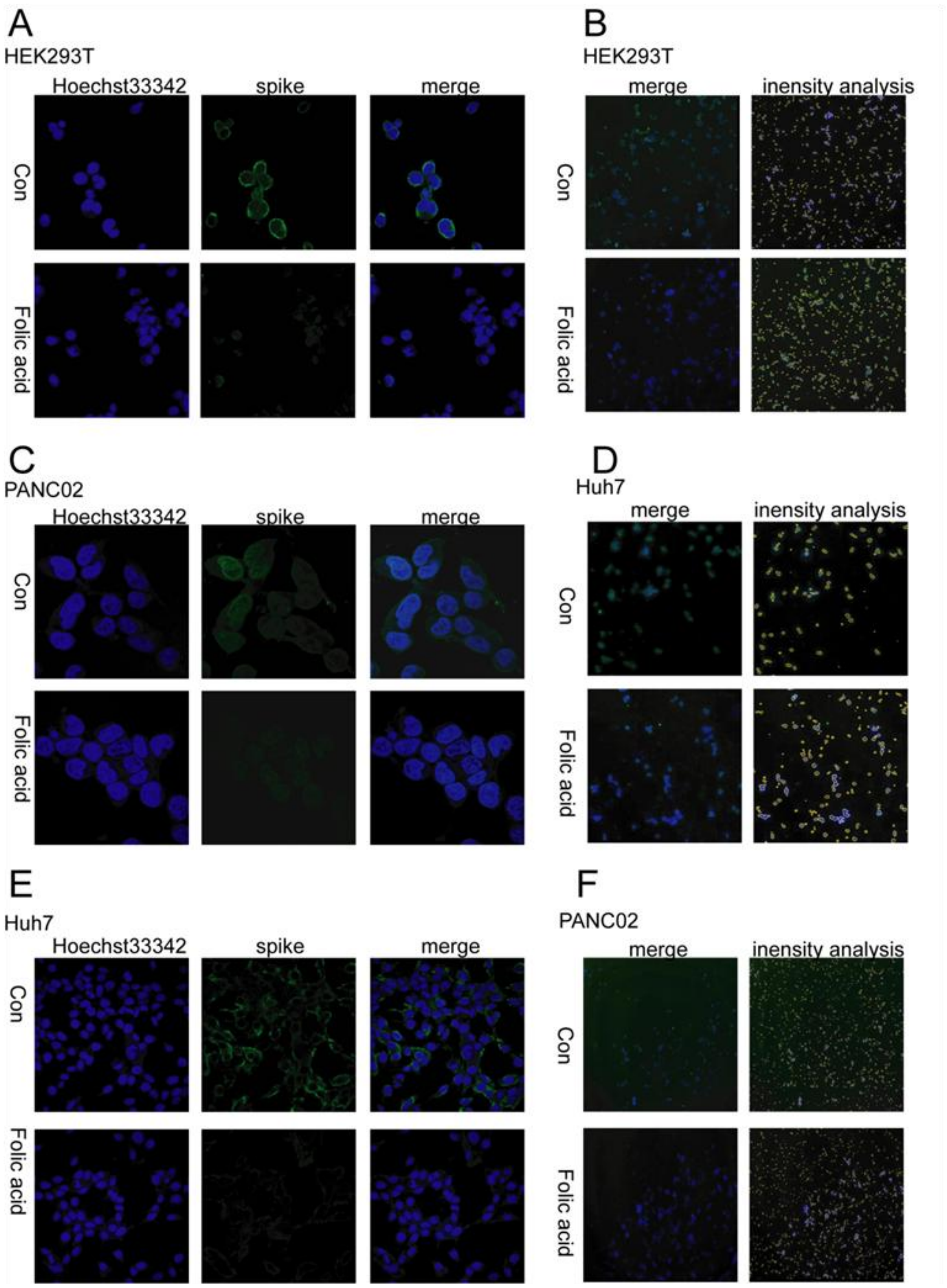

Supplementary Figure **3**. Folic acid treatment reduces the Spike protein binding ability. Immunofluorescence staining for spike protein in HEK293T (A) PANC02 (C) and Huh7 (E) cells. Fluorescence intensity quantitative analysis of Hoechst and spike signals in HEK293T (B) PANC02 (D) and Huh7 (F) cells by Celigo.

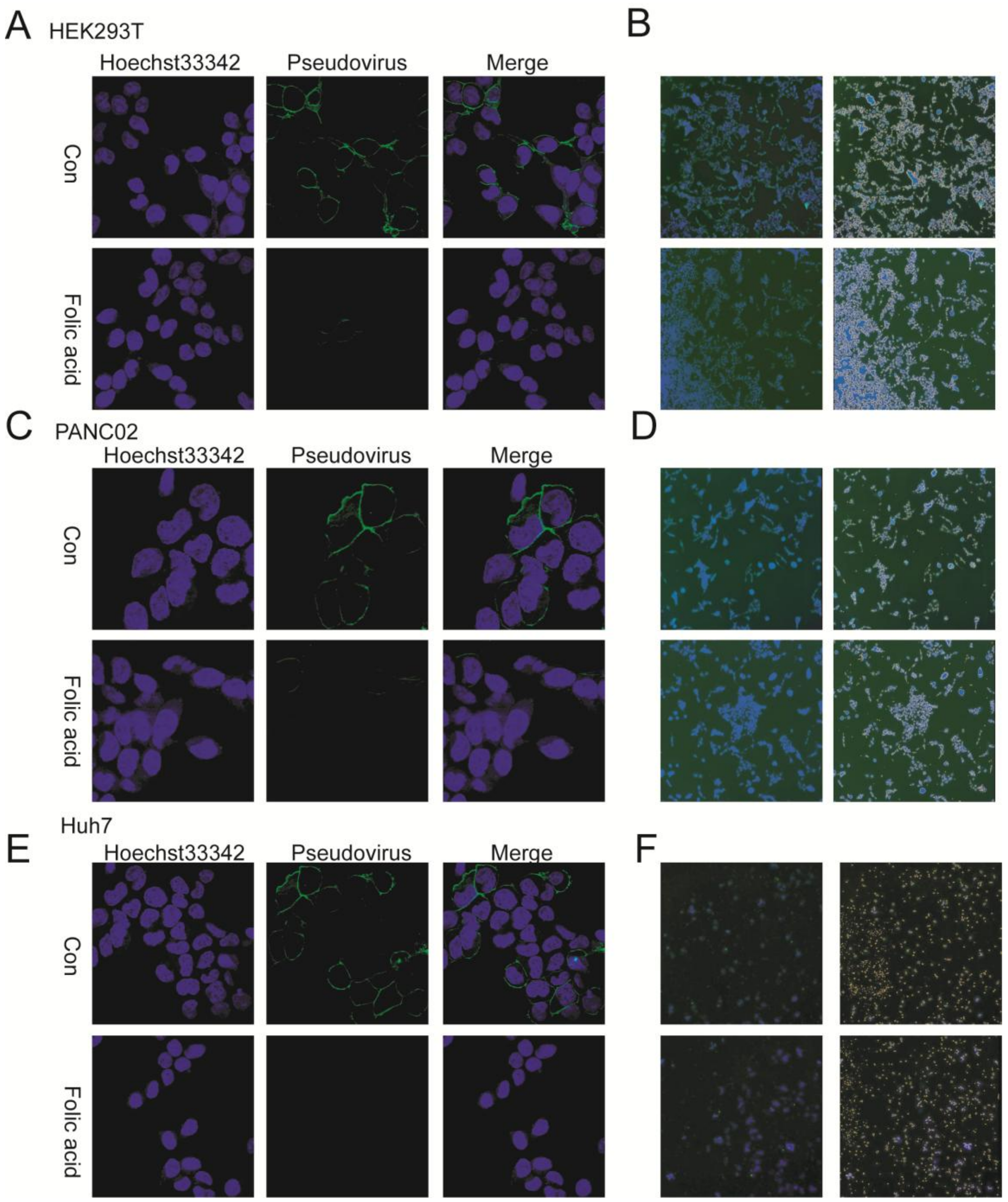

Supplementary Figure **4**. Folic acid treatment weakened the binding ability of pseudovirus. Immunofluorescence staining for pseudovirus in HEK293T (A) PANC02 (C) and Huh7 (E) cells. Immunofluorescence staining for pseudovirus and DIR in HEK293T (B) PANC02 (D) and Huh7 (F) cells.

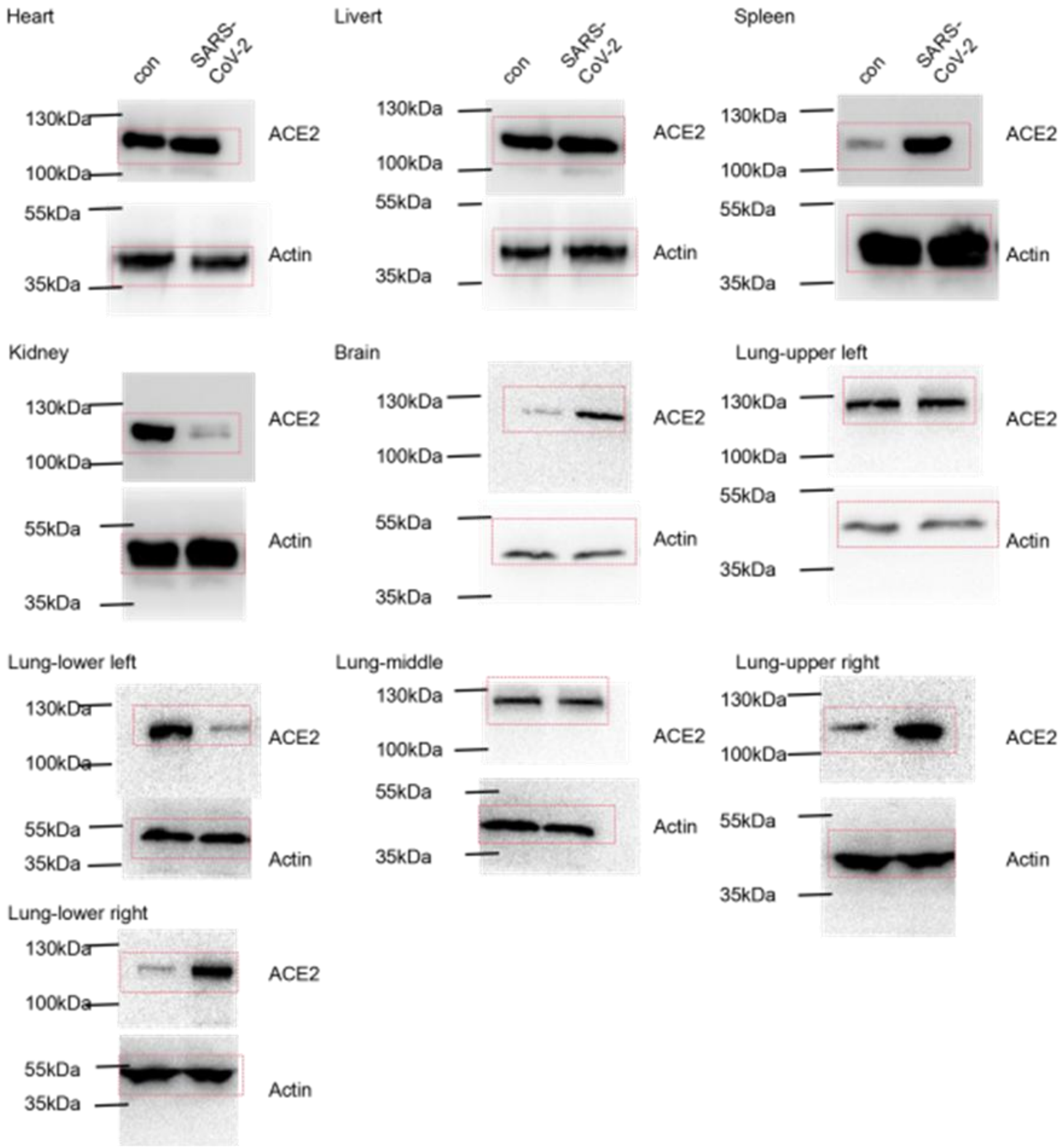

Supplementary Figure 5
Unedited western blot images corresponding to the gel images in Figure 1D.

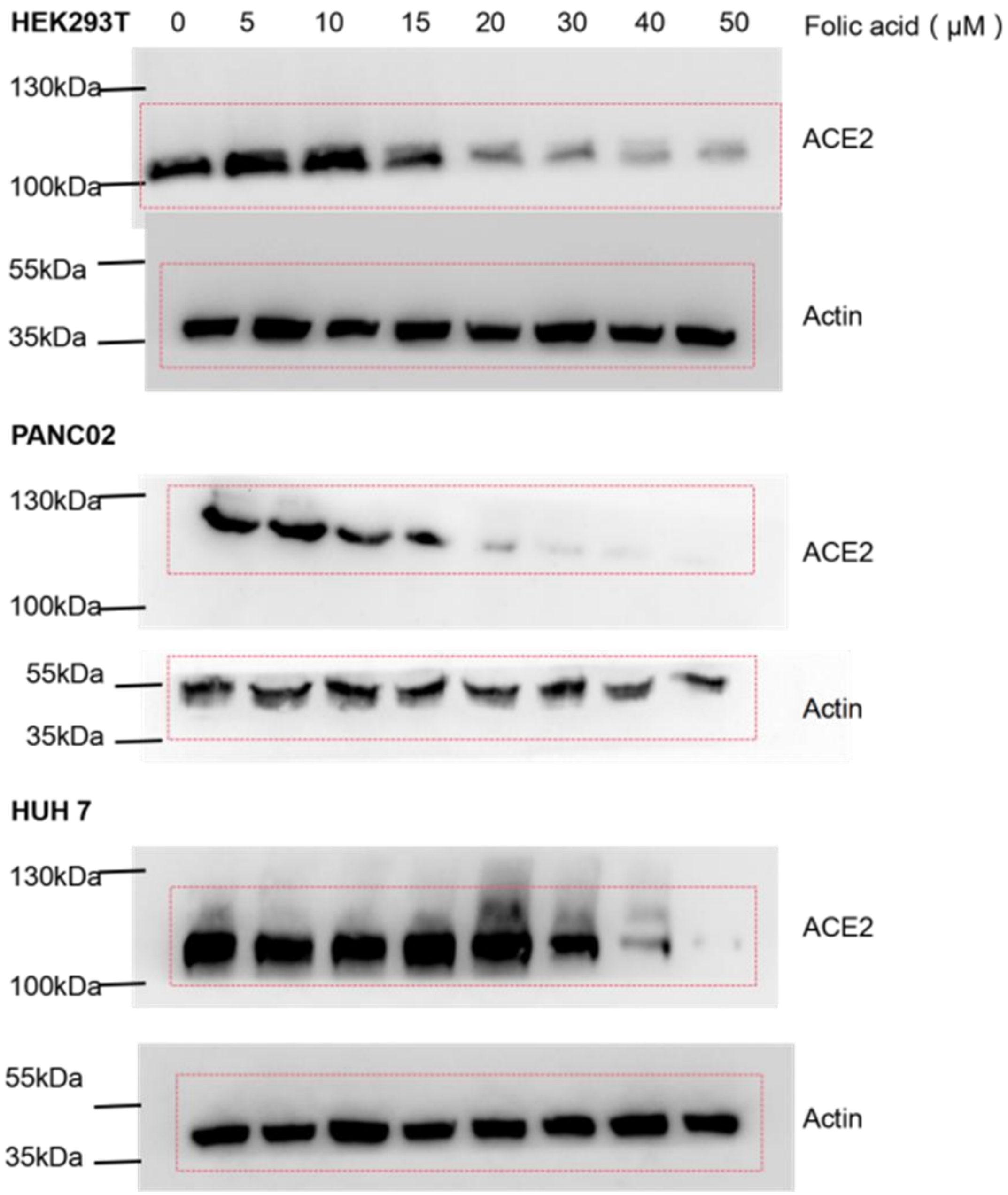

Supplementary Figure 6

Unedited western blot images corresponding to the gel images in Figure 2B.

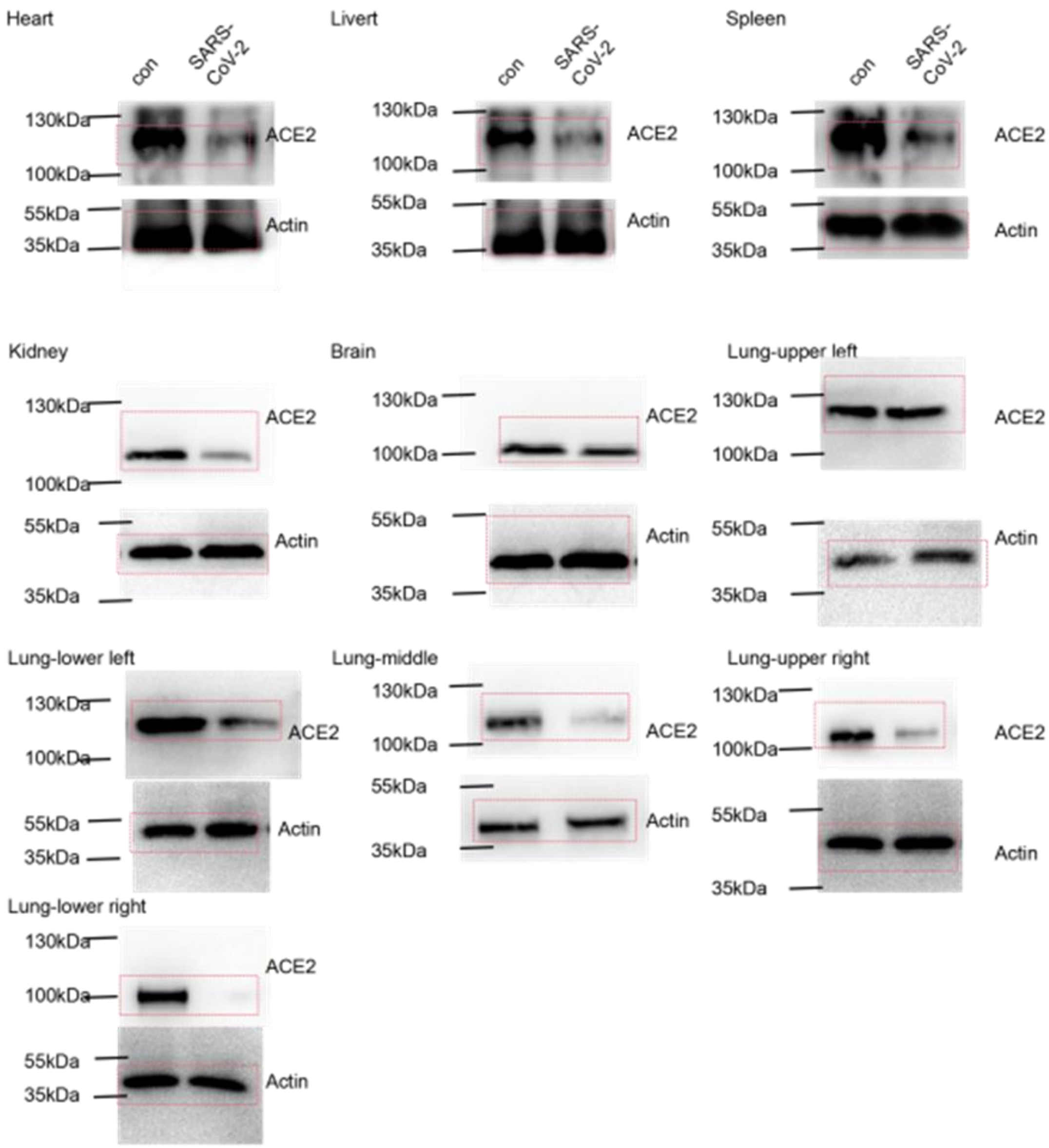

Supplementary Figure 7

Unedited western blot images corresponding to the gel images in Figure 4A.

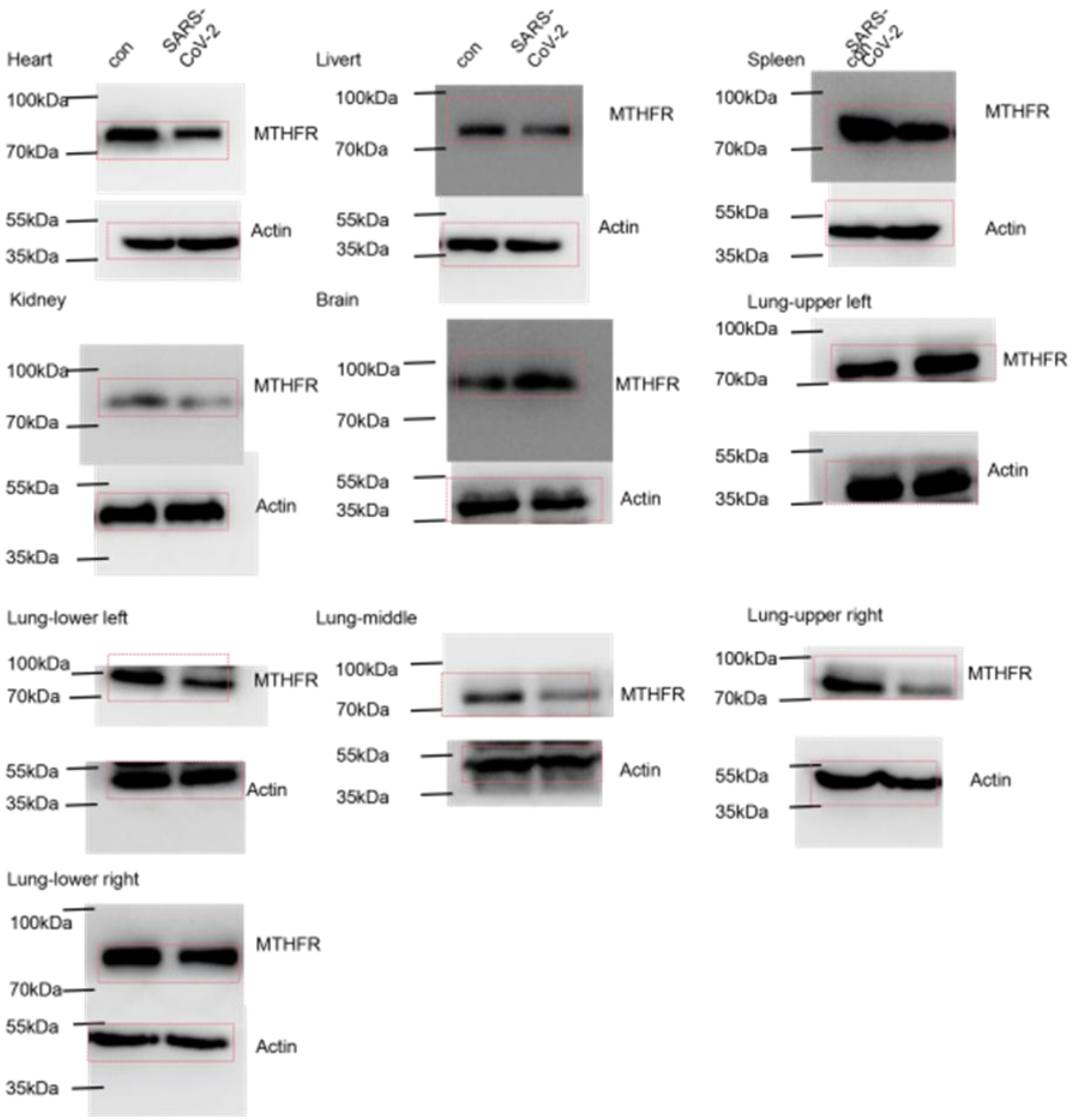

Supplementary Figure 8

Unedited western blot images corresponding to the gel images in Supplementary Figure 1G.
